# A new method of finger / thumb reconstruction using composite tissue

**DOI:** 10.1186/1753-6561-9-S3-A62

**Published:** 2015-05-19

**Authors:** Zengtao Wang

**Affiliations:** 1 Department of Hand and Foot Surgery, Shandong University College of Medicine, Shandong, 250021, China

## 

This article presents authors’ methods of digital reconstruction using composite tissue transfer. The authors present their approach to achieve the goal of restoring full cosmetic appearance of the reconstructed toes while preserving the function and cosmetic appearance of the donor foot. The reconstructive procedures for each degree of digit defect are discussed in detail, and pitfalls and technical tips are given. The article is a summary of the authors’ experience in reconstruction of 646 digits since 1998 and the challenges we faced in the complex microsurgical reconstruction necessary in pursuing the goal of restoring the cosmetic appearance of reconstructed digits and donor feet.

1. We present our methods of digital reconstruction using composite tissue transfer.

2. Our major aims of surgery are to improve the cosmetic appearance of reconstructed hands and donor feet.

3. We describe a number of methods using modified or novel designs for composite tissue transfer from the foot to improve cosmetic appearance of the reconstructed digits and to improve both the cosmetic appearance and function of donor feet.

4. The article provides technical details of the methods we used to move closer to an ideal reconstruction of digits and minimizing donor foot morbidity.

